# Birth by Caesarean Section and Prevalence of Risk Factors for Non-Communicable Diseases in Young Adults: A Birth Cohort Study

**DOI:** 10.1371/journal.pone.0074301

**Published:** 2013-09-09

**Authors:** Bernardo L. Horta, Denise P. Gigante, Rosangela C. Lima, Fernando C. Barros, Cesar G. Victora

**Affiliations:** 1 Postgraduate Program in Epidemiology, Universidade Federal de Pelotas, Pelotas, Rio Grande do Sul, Brazil; 2 Universidade Federal da Grande Dourados, Dourados, Mato Grosso do Sul, Brazil; 3 Postgraduate Program in Health and Behavior, Catholic University of Pelotas, Pelotas, Rio Grande do Sul, Brazil; Aga Khan University, Pakistan

## Abstract

**Background:**

Conflicting findings on the risk of obesity among subjects born by caesarean section have been published. Caesarean section should also increase the risk of obesity related cardiovascular risk factors if type of delivery is associated with obesity later in life. This study was aimed at assessing the effect of type of delivery on metabolic cardiovascular risk factors in early adulthood.

**Methodology and Principal Findings:**

In 1982, maternity hospitals in Pelotas, southern Brazil, were visited and those livebirths whose family lived in the urban area of the city have been followed. In 2000, when male subjects undertook the Army entrance examination (n=2200), fat mass and fat free mass were estimated through bioimpedance. In 2004–2005, we attempted to follow the whole cohort (n=4297), and the following outcomes were studied: blood pressure; HDL cholesterol; triglycerides; random blood glucose, C-reactive protein, waist circumference and body mass index. The estimates were adjusted for the following confounders: family income at birth; maternal schooling; household assets index in childhood; maternal skin color; birth order; maternal age; maternal prepregnancy weight; maternal height; maternal smoking during pregnancy; birthweight and family income at early adulthood.

**Results:**

In the crude analyses, blood pressure (systolic, diastolic and mean arterial pressure) and body mass index were higher among subjects who were delivered through caesarean section. After controlling for confounders, systolic blood pressure was 1.15 mmHg (95% confidence interval: 0.05; 2.25) higher among subjects delivered by caesarean section, and BMI 0.40 kg/m^2^ (95% confidence interval: 0.08; 0.71). After controlling for BMI the effect on systolic blood pressure dropped to 0.60 mmHg (95% confidence interval: -0.47; 1.67). Fat mass at 18 years of age was also higher among subjects born by caesarean section.

**Conclusion:**

Caesarean section was associated with a small increased in systolic blood pressure, body mass index and fat mass.

## Introduction

There is growing evidence that the development of non-communicable diseases may be programmed by exposures in early life [1,2]. Birthweight and weight gain in childhood have been the most commonly assessed exposures, but little attention has been given to the process of birth, i.e., the type of delivery [3].

Caesarean rates have shown upward trends in many countries, thus raising concerns on potential long-term consequences [4–6]. Studies from developed countries suggest that subjects born by caesarean section present increased risk of immune-related diseases in later life such as allergies [7], asthma [8], celiac disease [9], and type 1 diabetes [10]. Differences in intestinal bacterial colonisation - caused by lack of contact with maternal vaginal and intestinal flora [11] - may account for the higher risk of immune related disorders. The intestinal flora of infants born through caesarean section is less diverse in terms of bacterial species, and Bifidobacteria spp. are absent [12,13]. Indeed, children with atopic dermatitis and those who were born by caesarean section have similar patterns of intestinal colonization [14,15].

Recent studies have also postulated that caesarean section increases the risk of obesity [16–18], although other authors have disputed these findings [19,20]. Three studies evaluated the prevalence of obesity in childhood [16,18,19], whereas other two were carried out in early adulthood [17,20]. If caesarean section indeed increases the risk of obesity, it is also likely to affect other metabolic and cardiovascular risk factors that are closely associated with obesity. To our knowledge, the potential effect of caesarean section on metabolic cardiovascular risk factors has not been studied. We investigated this association in a population-based birth cohort study with 23 years of follow-up in Brazil, in which over a quarter of all infants were delivered by caesarean sections.

## Methods

In 1982, the five maternity hospitals in Pelotas, a southern Brazilian city (current population 350,000), were visited daily. The 5,914 liveborns whose families lived in the urban area were examined and their mothers interviewed when data on the gestation, birth and socioeconomic conditions was gathered. There were less than 1% of refusals at recruitment. This cohort has been followed up on several occasions [21]. Newborns were weighed soon after birth with regularly calibrated paediatric scales (precision 10 g) and gestational age was assessed based on the last menstrual period. Babies were classified as small for gestational age (SGA) or otherwise, using the 10^th^ centile of the Williams curves [22].

From October 2004 to August 2005, a city census was carried out in search of subjects belonging to the cohort. Those who were not located during the census were sought at their most recent available address. Subjects answered a questionnaire and were examined at home, and then invited to visit the research laboratory to donate a blood sample, collected by venous puncture.

The following metabolic risk factors of cardiovascular diseases were assessed:

- Blood pressure: blood pressure was measured at the beginning and at the end of the interview, using a digital wrist sphygmomanometer (Omron HEM-629). The mean values were used for most analyses. Mean arterial pressure was calculated as 1/3 (systolic blood pressure) + 2/3 (diastolic blood pressure) [23]. We also analyzed systolic and diastolic pressure separately.
- HDL cholesterol was measured using an ultrasensitive direct method, with a Selectra 2 analyzer (Merck).
- Triglyceride was assessed with a colorimetric enzymatic method.
- Random blood glucose: was assessed from fingertip blood, at the time of collecting the blood samples, using a portable glucose meter (Accu-Check Advantage – Roche). Because glucose levels vary according to fasting time, we adjusted the glucose levels for time since the last meal using linear regression models with glucose as the dependent variable, and time since the last meal as independent variable [24].
- C-reactive protein (CRP): was measured using an Immulite chemiluminescent immunoassay (Siemens). Because the lower detection limit was 0.1 mg/l, measures below that value were converted to 0.05 mg/l. Subjects with CRP >10 mg/l (n=198) were excluded from the analyses involving CRP, because such levels usually indicate acute conditions. Pregnant women (n=93) and those using oral contraceptives (n=445) were also excluded from the analyses involving CRP because they reportedly present elevated CRP levels [25].
- Body composition: was assessed at 18 years when males subject undertook the Army entrance examination in year 2000 (n=2200). Fat mass and fat free mass were estimated through bioimpedance using a Tanita Body Fat Analyser scale (model TBF -305; Tokyo, Japan). A validation study using total body water assessed through deuterium dilution method as the gold standard was carried out among 48 subjects in the same age group of the studied population [26]. Based on the information from this study, bioimpedance equations for fat mass (TBW/0.732) and lean mass (weight mass – fat mass) were corrected. Body composition outcomes included fat mass and fat free mass indices, both divided by height squared and expressed as kg/m^2^ [26].

Potential confounding factors included socioeconomic position and other maternal characteristics that are known to be associated with the risk of caesarean section. Information on confounding variables was collected in the early phases of the study. These included family income at birth in multiples of 1982 minimum wage (with one minimum wage being equivalent to US$50 a month in 1982), maternal schooling, household assets index (obtained through factor analysis and based on the ownership of household goods), maternal skin color, birthweight, maternal age, birth order, maternal smoking during pregnancy, maternal prepregnancy weight and height, and family income at early adulthood.

Triglycerides and CRP values (mg/L) were natural log-transformed (lnmg/L) for greater symmetry prior to undertaking statistical analyses. In the crude analysis, means were compared using analysis of variance. Adjusted analyses, controlling for the above listed confounders, were carried out using linear regression analysis. Statistical comparisons between groups were based on tests of heterogeneity and linear trend in the case of ordinal variables, and the one with the lower p-value was presented.

The Ethical Review Board of the Faculty of Medicine of the Federal University of Pelotas approved the study, and written informed consent was obtained from participating subjects in 2004-5. In 1982, verbal consent was obtained from the mothers, as this was the standard practice at that time. The results of biomarkers were returned to the cohort member and if necessary the subjects were referred to the university health service

## Results

In the 2004-5 follow-up visit, 4297 subjects were interviewed; adding the 282 individuals who were known to have died, this represented a follow-up rate of 77.4%. Table 1 shows that follow-up rate in the 2004-5 visit was not related to birthweight, sex or type of delivery. On the other hand, attrition rate was higher among those subjects whose family income was at either the upper or lower end of distribution and those whose mother had 12 or more years of schooling. In 2004-5, 3914 subjects agreed to provide blood samples, and caesarean section was also not associated with the proportion of subjects who donated a blood sample. In the 2000 visit, maternal schooling was not associated with attrition rate, whereas for family income, the association was similar to that observed in the 2004-5 visit. As for 2004-5, type of delivery and birthweight were not associated with the proportion of subjects who were followed-up.

**Table 1:** Follow-up rate in different phases of the study according to some characteristics of the cohort.

	Percent located		
	2000 visit	2004-5 visit	2004-5 visit and donated blood
**Gender**			
Male	78.9	78.2	65.0
Females	−	76.6	67.5
**Family income at birth in multiples of 1982 minimum wages**			
≤ 1	72.7	74.7	61.2
1.1-3	80.1	80.8	70.3
3.1-6	84.0	76.0	67.2
6.1-10	79.4	68.3	56.0
> 10	76.7	74.0	59.7
**Maternal schooling at birth (in years)**			
≤ 4	76.2	78.4	66.6
5-8	81.7	79.2	68.5
9-11	75.8	75.8	64.5
≥ 12	79.6	71.0	59.8
**Birthweight in grams**			
< 2500	77.5	77.9	52.8
2500-2999	78.1	78.3	67.4
3000-3499	78.7	76.2	66.9
3500-3999	80.2	79.8	70.5
≥ 4000	79.9	71.6	60.3
**Type of delivery**			
Vaginal	77.7	77.2	66.2
Caesarean section	82.1	77.9	66.2
**Number of subjects evaluated**	2250	4297	3914

Includes subjects interviewed as well as those who are known to have died;

Table 2 shows that among those subjects included in the present analyses, the prevalence of caesarean section was 28.2% for males and 27.8% for females, compared to 27.6% in the original cohort. For males, mean birthweight was 3279 g; 15.1% of the subjects were born small for gestational age, whereas 5.5% were preterm, whereas for females the prevalence of small for gestational age and preterm was 13.8% and 5.2%, respectively. At 23 years, mean body mass index (BMI) was 23.8 and 23.4 kg/m^2^, respectively, for males and females.

**Table 2:** Distribution of sample studied at 23 years of age, according to key characteristics.

Sample characteristics		Males			Females		
		N	Mean (SD)	Prevalence (%)	N	Mean (SD)	Prevalence (%)
**At birth**							
Birthweight (g)		2208	3279 (523)		2082	3162 (503)	
Preterm birth		97		5.5	86		5.2
Small-for-gestational age		268		15.1	229		13.8
Type of delivery							
	Vaginal	1586		71.8	1504		72.2
	Caesarean	622		28.2	579		27.8
**At 18 years**							
Fat mass over height^2^ (kg/m^2^)		2197	3.75 (1.5)				
Fat free mass over height^2^ (kg/m^2^)		2197	18.6 (2.3)				
**At 23 years**							
Triglycerides (mg/dL) #		1918	97.4 (78)		1906	85.8 (56)	
HDL cholesterol (mg/dL)		1918	51.6 (11.2)		1906	59.4 (13.5)	
Non-fasting glucose (mg/dL)		1863	99.8 (15.6)		1869	94.8 (14.1)	
Mean systolic blood pressure (mmHg)		2208	123.5 (14.4)		2083	111.1 (13.0)	
Mean diastolic blood pressure (mmHg)		2208	75.7 (11.6)		2083	71.3 (10.8)	
Mean arterial pressure (mmHg)		2208	91.6 (11.9)		2083	84.6 (11.1)	
C-reactive protein		1839	0.78 (1.40))		1252	1.31 (2.76)	
Waist circumference (cm)		2172	80.9 (10.1)		1934	74.7 (10.5)	
Mean body mass index (kg/m^2^)		2173	23.8 (4.1)		1935	23.4 (4.6)	

# Median (Interquartile range)

Table 3 shows the prevalence of caesarean section according to potential confounding variables. Socioeconomic position and maternal age were positively and strongly associated with the prevalence of caesarean section. Subjects born with a birthweight over 4 kg were more likely to have been delivered through a caesarean section.

**Table 3:** Prevalence of caesarean section according to socioeconomic status, demographic variables, birthweight and birth order.

	Prevalence of caesarean section (%)
**Family income at birth in multiples of 1982 minimum wages**	P < 0.001*
≤ 1	17.0
1.1-3	25.1
3.1-6	36.3
6.1-10	41.4
> 10	46.6
**Maternal schooling at birth (in years)**	P < 0.001*
≤ 4	19.9
5-8	26.1
9-11	33.6
≥ 12	45.4
**Maternal skin color**	P < 0.001
White	28.9
Black or mixed	21.5
**Household score asset index in quintiles**	P < 0.001*
1^st^	18.3
2^nd^	22.3
3^rd^	30.6
4^th^	32.1
5^th^	37.8
**Maternal age (in years)**	P < 0.001*
< 20	22.3
20-29	25.4
≥ 30	35.7
**Birthweight in grams**	P = 0.002 @
< 2500	27.0
2500-2999	26.6
3000-3499	26.1
3500-3999	28.9
≥ 4000	36.2
**Birth order**	P < 0001 @
1^st^	30.3
2^nd^	26.1
3^rd^	30.4
4^th^	20.9

* test for linear trend

@ test for heterogeneity

Table 4 shows the associations between early life socioeconomic position and the studied outcomes, stratified by sex. Systolic blood pressure and blood glucose were not associated with family income or maternal schooling at birth. Triglycerides and HDL cholesterol were positively associated with socioeconomic variables for males and females. On the other hand, BMI was positively associated with socioeconomic position among males, whereas for females the association was in the opposite direction.

**Table 4:** Metabolic cardiovascular risk factors according to family income and maternal schooling at birth, stratified by sex.

	Mean systolic blood pressure in mmHg (SE)	Mean diastolic blood pressure in mmHg (SE)	Mean triglycerides in mg/dL (SE) #	Mean HDL cholesterol in mg/dL (SE)	Mean non-fasting glucose in mg/dL (SE)	Mean body mass index in kg/m^2^ (SE)
**MALES**						
**Family income at birth in multiples of 1982 minimum wages**	P = 0.31 @	P = 0.22 @	P < 0.001*	P < 0.001 @	P = 0.43 @	P < 0.001*
≤ 1	123.2 (0.69)	75.9 (0.57)	88.6 (1.03)	51.9 (0.58)	100.0 (0.83)	23.2 (0.18)
1.1-3	123.7 (0.44)	75.6 (0.36)	97.8 (1.02)	50.8 (0.36)	99.3 (0.47)	23.7 (0.12)
3.1-6	123.2 (0.70)	75.3 (0.55)	102.4 (1.03)	51.6 (0.61)	100.6 (0.97)	24.3 (0.21)
6.1-10	124.4 (1.22)	77.9 (0.98)	105.1 (1.06)	55.2 (1.02)	100.7 (1.65)	24.2 (0.38)
> 10	123.0 (1.27)	75.0 (1.02)	102.9 (1.06)	53.9 (1.02)	98.1 (1.38)	24.8 (0.36)
**Maternal schooling at birth (in years)**	P = 0.49 @	P = 0.64 @	P = 0.001*	P = 0.05*	P = 0.18 @	P < 0.001*
≤ 4	123.7 (0.55)	75.8 (0.45)	93.3 (1.02)	51.1 (0.46)	98.8 (0.56)	23.4 (0.14)
5-8	123.0 (0.46)	75.4 (0.38)	96.9 (1.02)	51.6 (0.38)	100.6 (0.60)	23.8 (0.13)
9-11	124.4 (0.88)	76.3 (0.70)	102.5 (1.04)	51.2 (0.76)	99.8 (1.11)	24.1 (0.26)
≥ 12	123.9 (0.84)	76.1 (0.65)	106.1 (1.04)	53.1 (0.69)	99.3 (0.88)	24.6 (0.27)
**FEMALES**						
**Family income at birth in multiples of 1982 minimum wages**	P = 0.94 @	P = 0.49 @	P < 0.001*	P < 0.001*	P = 0.93 @	P < 0.001*
≤ 1	111.1 (0.69)	71.2 (0.59)	81.9 (1.02)	56.9 (0.66)	94.8 (0.77)	23.7 (0.27)
1.1-3	111.3 (0.41)	71.0 (0.34)	85.6 (1.02)	58.3 (0.42)	94.7 (0.44)	23.6 (0.15)
3.1-6	111.2 (0.61)	72.2 (0.49)	89.5 (1.03)	62.0 (0.73)	95.0 (0.79)	23.3 (0.22)
6.1-10	110.4 (1.16)	70.9 (0.93)	86.7 (1.05)	63.3 (1.40)	94.4 (1.46)	22.7 (0.33)
> 10	110.6 (1.18)	71.3 (0.94)	90.4 (1.05)	67.3 (1.37)	96.0 (1.44)	21.6 (0.25)
**Maternal schooling at birth (in years)**	P = 0.30*	P = 0.02*	P = 0.005*	P < 0.001*	P = 0.17*	P < 0.001*
≤ 4	110.9 (0.51)	70.7 (0.43)	84.5 (1.02)	57.1 (0.50)	95.2 (0.58)	23.9 (0.20)
5-8	111.1 (0.44)	71.3 (0.36)	84.1 (1.02)	58.8 (0.47)	94.8 (0.49)	23.5 (0.17)
9-11	111.4 (0.83)	72.0 (0.73)	89.2 (1.03)	61.3 (1.00)	94.7 (0.96)	22.8 (0.26)
≥ 12	111.8 (0.74)	72.2 (0.57)	93.0 (1.03)	65.7 (0.84)	93.7 (0.92)	22.2 (0.19)

* test for linear trend

@ test for heterogeneity

# geometric mean

Analyses were initially stratified by sex (Tables 5 and 6). In the crude analyses, there was evidence for increased blood pressure, waist circumference and BMI among males who were delivered through caesarean section. Among women, there were no significant differences. We tested for interactions between caesarean section and sex for all outcomes. For waist circumference (P for interaction = 0.02), there was a positive association with caesarean sections among males and females, and the confidence intervals included the reference for both estimates. For blood glucose, there was a positive association for men and a negative one for women, and the confidence intervals included the reference(P for interaction = 0.13). For CRP (P for interaction = 0.07) there was a stronger positive association with caesarean section for women. Interactions for the remaining outcomes had P levels of 0.19 or greater. Data on fat and fat-free mass indices were available for eighteen-year-old males. The average fat mass index was 3.67 kg/m^2^ for those delivered vaginally compared to 3.93 kg/m^2^ for those delivered by caesarean section (p-value < 0.001). Adjusted analyses are shown in Table 6. The first set of adjusted models included confounding variables, and the second set also includes adjustment for BMI, except when BMI, waist circumference, fat mass or fat free mass were the outcome. In general, associations among males were attenuated by adjustment for confounding; all adjusted confidence intervals except for fat mass included the value of zero (for linear regression analyses of blood pressure, HDL cholesterol, waist circumference, BMI and fat free mass) or one (for analyses of log-triglycerides or CRP). Among females, for some of the risk factors, confounding was negative, i.e., tended to underestimate the magnitude of the association and adjustment for confounders increased the magnitude of the regression coefficient. For BMI, adjusted regression coefficient for females increased from 0.27 to 0.47 kg/m^2^. As was the case in the crude analyses, the only significant interaction was for waist circumference (p=0.02) but the interaction for CRP had a p value of 0.07.

**Table 5:** Metabolic cardiovascular risk factors according to the type of delivery.

	Males			Females			p-value for interaction
	Type of delivery		p-value	Type of delivery		p-value	
	Vaginal	Caesarean section		Vaginal	Caesarean section		
Mean systolic blood pressure (SD)	123.1 (14.4)	124.5 (14.3)	0.04	111.0 (13.0)	111.5 (13.2)	0.42	0.38
Mean diastolic blood pressure (SD)	75.4 (11.6)	76.5 (11.6)	0.04	71.1 (10.8)	71.7 (10.8)	0.26	0.49
Mean of mean arterial pressure in mmHg (SD)	91.3 (11.9)	92.5 (11.9)	0.03	84.4 (11.0)	85.0 (11.1)	0.29	0.42
Mean non-fasting blood glucose in mg/dL (SD)	99.5 (15.8)	100.4 (15.2)	0.25	95.0 (14.4)	94.2 (13.3)	0.22	0.10
Mean HDL cholesterol in mg/dL (SD)	51.5 (11.4)	51.8 (10.8)	0.52	59.2 (13.7)	59.9 (12.7)	0.35	0.76
Mean triglycerides in mg/dL (SD) #	96.4 (1.75)	99.9 (1.84)	0.23	85.4 (1.62)	86.9 (1.60)	0.48	0.65
Mean C reactive protein (SD)#	0.79 (3.2)	0.77 (3.1)	0.74	1.26 (3.4)	1.45 (3.1)	0.07	0.10
Mean waist circumference in cm (SD)	80.5 (10.1)	81.9 (9.9)	0.003	74.8 (10.7)	74.6 (9.9)	0.71	0.02
Mean body mass index in kg/m^2^ (SD)	23.6 (4.1)	24.3 (4.0)	< 0.001	23.3 (4.6)	23.6 (4.6)	0.25	0.15
Fat mass over height^2^ in kg/m^2^ (SD)	3.67 (1.5)	3.93 (1.5)	< 0.001				
Fat free mass over height^2^ (kg/m^2^)	18.5 (2.3)	18.9 (2.3)	< 0.001				

Unadjusted analyses.

# geometric mean

**Table 6:** Adjusted regression coefficients for metabolic cardiovascular risk factors at 23 years according to type of delivery.

	Regression coefficients comparing subjects born by caesarean section in relation to vaginal delivery (95% confidence interval)						p-value for interaction in confounder-adjusted models
	MALES			FEMALES			
	Crude	Adjusted for confounders^#^	Adjusted for confounders + BMI	Crude	Adjusted for confounders^#^	Adjusted for confounders + BMI	
Systolic blood pressure in mmHg	1.34 (0.01; 2.67) n = 2208	1.30 (-0.16; 2.75) n = 1983	0.77 (-0.62; 2.15) n = 1948	0.52 (-0.73; 1.77) n = 2083	0.74 (-0.65; 2.14) n = 1891	0.16 (-1.20; 1.52) n = 1758	0.47
Diastolic blood pressure in mmHg	1.12 (0.05; 2.21) n = 2208	0.86 (-0.32; 2.04) n = 1983	0.50 (-0.63; 1.64) n = 1948	0.60 (-0.44; 1.63) n = 2083	0.29 (-0.87; 1.44) n = 1891	0.04 (-1.09; 1.18) n = 1758	0.47
Mean arterial pressure in mmHg	1.20 (0.09; 2.30) n = 2208	1.00 (-0.20; 2.21) n = 1983	0.59 (-0.56; 1.74) n = 1948	0.57 (-0.49; 1.63) n = 2083	0.44 (-0.75; 1.62) n = 1891	0.08 (-1.08; 1.24) n = 1758	0.45
Non-fasting blood glucose in mg/dL	0.92 (-0.66; 2.50) n = 1863	0.71 (-1.02; 2.44) n = 1676	0.74 (-0.99; 2.48) n = 1648	-0.90 (-2.35; 0.54) n = 1869	-0.87 (-2.50; 0.75) n = 1692	-1.23 (-2.87; 0.42) n = 1568	0.13
HDL cholesterol in mg/dL	0.37 (-0.75; 1.48) n = 1918	-0.23 (-1.44; 0.98) n = 1726	0.00 (-1.21; 1.23) n = 1696	0.65 (-0.71; 2.01) n = 1906	-1.70 (-3.16; -0.24) n = 1731	-1.39 (-2.86; 0.07) n = 1607	0.6
Log- Triglycerides in mg/dL	1.04 (0.98; 1.10) n = 1918	1.01 (0.95; 1.07) n = 1726	1.00 (0.94; 1.05) n = 1696	1.02 (0.97; 1.07) n -1906	1.00 (0.95; 1.06) n = 1731	0.99 (0.94; 1.05) n = 1607	0.63
Log -C reactive protein	0.98 (0.87; 1.10) n = 1839	0.93 (0.82; 1.06) n = 1656	0.90 (0.79; 1.01) n = 1628	1.14 (0.99; 1.33) n = 1252	1.20 (1.01; 1.41) n = 1143	1.13 (0.96; 1.34) n = 1111	0.07
Waist circumference in cm	1.44 (0.49; 2.38) n = 2172	0.74 (-0.25; 1.73) n = 1950		-0.20 (-1.24; 0.84) n = 1934	0.56 (-0.54; 1.67) n = 1757		0.02
Body mass index in kg / m^2^	0.70 (0.32; 1.08) n = 2173	0.37 (-0.03; 0.77) n = 1951		0.27 (-0.19; 0.73) n = 1935	0.47 (-0.02; 0.96) n = 1758		0.19
Fat mass over height^2^ in kg/m^2^	0.26 (0.12; 0.40) n = 2197	0.17 (0.01; 0.33) n = 1775					
Fat free mass over height^2^	0.37 (0.15; 0.58) n = 2197	0.13 (-0.11; 0.38) n = 1775					

# Adjusted for: Family income at birth, maternal schooling at birth, household assets index in childhood, maternal skin color, birth order, maternal age, maternal prepregnancy weight, maternal height, maternal smoking during pregnancy, birthweight, and family income in early adulthood.

Because no interactions were detected for most variables, we present the pooled models in Table 7. In the unadjusted analyses, blood pressure (systolic, diastolic and mean arterial pressure) and body mass index were higher among those subjects who were born by caesarean section. However, blood glucose, HDL cholesterol, triglycerides, waist circumference and CRP were not associated with type of delivery. Even after controlling for possible confounding variables, mean body mass index at 23 years was higher among those subjects who were born by caesarean section. On the other hand, the difference in mean arterial pressure between subjects born by caesarean section and vaginal delivery was slightly reduced in the multivariate model from 0.93 to 0.80 mmHg and the confidence interval included the null value (95% confidence interval: -0.08; 1.68). But for systolic blood pressure, the difference slightly increased from 1.00 to 1.15mmHg and the confidence interval did not include the null value (95% confidence interval: 0.05; 2.25). After adjusting for body mass index at 23 years, a possible mediator, the difference in mean arterial pressure was further reduced to 0.42 mmHg (95% confidence interval: -0.43; 1.27). For systolic blood pressure the difference was also reduced after controlling to body mass index. [mean difference: 0.60 mmHg (95% confidence interval: -0.47; 1.67)]. In the multivariate analysis, HDL cholesterol was slightly lower among those subjects born by caesarean section, but the confidence interval included the reference. As for blood pressure, the regression coefficient for HDL was reduced after controlling for body mass index. For fat mass, the beta coefficient in the unadjusted linear regression equation was equal to 0.26 kg/m^2^ (95% confidence interval: 0.12; 0.40); after adjustment for confounders the value dropped to 0.17 kg/m^2^ (95% confidence interval: 0.01; 0.33). The corresponding betas for the fat free mass index were 0.37 kg/m^2^ (95% confidence interval: 0.15; 0.58) and 0.13 kg/m^2^ (95% confidence interval: -0.11; 0.38).

**Table 7:** Adjusted regression coefficients for metabolic cardiovascular risk factors at 23 years according to type of delivery.

	Regression coefficients comparing subjects born by caesarean section in relation to vaginal delivery (95% confidence interval)		
	Crude	Adjusted for confounders^#^	Adjusted for confounders + BMI
Systolic blood pressure in mmHg	1.00 (0.00; 2.00) n = 4291	1.15 (0.05; 2.25) n = 3874	0.60 (-0.47; 1.67) n = 3706
Diastolic blood pressure in mmHg	0.89 (0.13; 1.66) n = 4291	0.63 (-0.21; 1.47) n = 3874	0.34 (-0.48; 1.15) n = 3706
Mean arterial pressure in mmHg	0.93 (0.13; 1.73) n = 4291	0.80 (-0.08;1.68) n = 3874	0.42 (-0.43; 1.27) n = 3706
Non-fasting blood glucose in mg/dL	0.08 (-1.01; 1.16) n = 3732	-0.02 (-1.22; 1.18) n = 3368	-0.20 (-1.41; 1.01) n = 3216
HDL cholesterol in mg/dL	0.44 (-0.49; 1.36) n = 3824	-0.96 (-1.96; 0.04) n = 3457	-0.63 (-1.62; 0.37) n = 3303
Log- Triglycerides in mg/dL	1.03 (0.99; 1.07) n = 3824	1.01 (0.97; 1.05) n = 3457	0.99 (0.96; 1.04) n = 3303
Log -C reactive protein	1.05 (0.95; 1.15) n = 3091	1.02 (0.92; 1.13) n = 2799	0.98 (0.89; 1.09) n = 2739
Waist circumference in cm	0.66 (-0.07; 1.38) n = 4106	0.60 (-0.18; 1.38) n = 3707	
Body mass index in kg / m^2^	0.50 (0.20; 0.79) n = 4108	0.40 (0.08; 0.71) n = 3709	
Fat mass over height^2^ in kg/m^2^ -18 years	0.26 (0.12; 0.40) n = 2197	0.17 (0.01; 0.33) n = 1775	
Fat free mass over height^2^ in kg/m^2^ -18 years	0.37 (0.15; 0.58) n = 2197	0.13 (-0.11; 0.38) n = 1775	

# Adjusted for: Family income at birth, maternal schooling at birth, household assets index in childhood, maternal skin color, birth order, maternal age, maternal prepregnancy weight, maternal height, maternal smoking during pregnancy, birthweight, and family income in early adulthood.

To sum up, in the confounder-adjusted models, systolic blood pressure was 1.15 mm Hg higher among subjects delivered by caesarean section, BMI was 0.40 kg/m^2^ high, and fat mass was.0.17 kg/m^2^ high. Adjustment for BMI reduced the effect on systolic blood pressure to 0.60 mmHg, with a confidence interval that includes zero. None of the other risk factors showed an association with caesarean sections.

## Discussion

In this cohort that has been prospectively followed since birth, there were weak but statistically significant associations between type of delivery and blood pressure and body mass index at 23 years, and with fat mass in males at 18 years of age. No other metabolic or cardiovascular risk factors were associated with type of delivery. There was heterogeneity by sex in the analyses of some risk factors, but not with those for which there was statistical evidence of an association.

Information on type of delivery was collected soon after birth, so that misclassification was unlikely. A limitation of the study was that blood glucose and lipids levels were obtained from non-fasting samples. However, fasting time was not related to type of delivery: the proportion of individuals who had fasted for eight or more hours was 10.7% among those who were born by caesarean section and 10.1% among those who were born by vaginal delivery. Therefore, differential misclassification is unlikely to have affected the present analyses. In addition, although triglyceride levels are affected by fasting time and time since last meal [27], current evidence suggests that non-fasting levels are a better predictor of cardiovascular risk than fasting levels [28,29].

The possibility of residual confounding must be taken into consideration in the assessment of the evidence from studies on long-term consequences of type of delivery. Because the prevalence of caesarean section is strongly and directly related to socioeconomic position, residual confounding may affect associations with any outcomes that also vary according to wealth. We examined how residual confounding might have affected the observed associations with blood pressure and BMI. Consistently with our earlier results [30] we found that blood pressure in early adulthood was not related to socioeconomic position. Therefore, adjustment for socioeconomic variables should have a small influence in the mean difference in blood pressure. Indeed, we observed that crude and adjusted mean differences were similar.

In our cohort, BMI is inversely associated with socioeconomic position in women, and directly associated in men [31]. In the same token, Monteiro et al [32] have observed that obesity should no more be considered as a problem of the rich and that the shift of obesity towards the poor happens initially among men. As expected from the pattern of confounding, adjustment attenuated the association with caesarean section in men and enhanced the association in women. The adjusted differences had similar magnitude, mean difference of 0.36 kg/m^2^ in males and 0.44 kg/m^2^ in females. This also suggests that residual confounding does not explain these results. Analyses of fat mass were restricted to males who had to undergo a medical examination in the Army. Adjustment for confounding reduced the magnitude of the association with type of delivery, but it still remained significant after adjustment.

Concerning selection bias, we followed-up 77.4% of the originally enrolled subjects in the 2004-5 visit. Because type of delivery was not related to follow-up rate, selection bias is unlikely to have affected the present results.

In terms of biological plausibility, it has been suggested that change in the gut microbiota might explain the effect of type of delivery on obesity [33]. Bifidobacteria spp. [12,13] are almost absent from faecal samples of infants who were born by caesarean section, and Kalliomäki et al [34] observed that children who became overweight at 7 years presented a lower quantity of Bifidobacteria at 6 months and 1 year of age than those whose weight remained normal. It was hypothesised that this change in gut microbiota would also be related to chronic inflammation. Furthermore, it has also been suggested that caesarean section could also affect hepatic and metabolic responses. Hyde et al [35] observed that steatosis was higher among piglets that were born by caesarean section. We did not observe any evidence of programming effect of type of delivery on blood lipids (triglycerides and HDL cholesterol) or CRP. Therefore, our findings do not support the hypothesis of programming of hepatic metabolism and inflammation by type of delivery.

A previously published study from our cohort failed to detect an association between type of delivery and prevalence of obesity at 23 years, using a cut-off of 30 kg/m^2^ [20]. In contrast, we analysed BMI as a continuous variable and found that mean values were higher among those subjects born by caesarean section. Two other studies have also reported that mean body mass index was higher among subjects who were born by caesarean section [16,17].

Our study investigated the associations between type of delivery and 11 different metabolic and cardiovascular risk factors. Three associations persisted after adjustment for confounders, but effect sizes were relatively small: 1.15 mmHg for systolic blood pressure, 0.40 kg/m^2^ for BMI, and 0.17 kg/m^2^ for fat mass index in males. Overall, although an effect of caesarean delivery on the risk of cardiovascular diseases may be present, this effect does not appear to be large except through its main causal pathway, body mass index.
